# Systematic searches for new inorganic materials assisted by materials informatics

**DOI:** 10.1080/14686996.2024.2428154

**Published:** 2024-11-11

**Authors:** Yukari Katsura, Masakazu Akiyama, Haruhiko Morito, Masaya Fujioka, Tohru Sugahara

**Affiliations:** a Center for Basic Research on Materials, National Institute for Materials Science (NIMS), Tsukuba, Japan; b Graduate School of Science and Technology, University of Tsukuba, Tsukuba, Japan; c RIKEN Center for Advanced Intelligence Project, RIKEN, Tokyo, Japan; d Faculty of Science, Academic Assembly, University of Toyama, Toyama, Japan; e Institute for Materials Research, Tohoku University, Sendai, Japan; f Innovative Functional Materials Research Institute, National Institute of Advanced Industrial Science and Technology (AIST), Nagoya, Japan; g Faculty of Materials Science and Engineering, Kyoto Institute of Technology, Kyoto, Japan

**Keywords:** Inorganic chemistry, materials informatics, machine learning, crystal structure analysis, database, high-throughput synthesis, ion diffusion control, autonomous measurement system, thermoelectric materials

## Abstract

We introduce our proprietary Materials Informatics (MI) technologies and our chemistry-oriented methodology for exploring new inorganic functional materials. Using machine learning on crystal structure databases, we developed ‘Element Reactivity Maps’ that displays the presence or the predicted formation probability of compounds for combinations of 80 × 80 × 80 elements. By analysing atomic coordinates with Delaunay tetrahedral decomposition, we established the concept of Delaunay Chemistry. This enabled us to design crystal structures by combining Delaunay tetrahedra of known compounds and to develop the ‘Crystal Cluster Simulator’ web system. We also developed the Starrydata2 web system to collect large-scale experimental data on material properties from plot images in academic papers. This dataset supported us to select candidate materials for new thermoelectric materials through various data analyses. In large-scale synthesis experiments involving over 7,000 samples, we discovered numerous new phases, including solid solutions of known structures in new combinations of elements. Using sodium metal in synthesis and our proprietary ion diffusion control technologies, we discovered new cage-like compounds by extracting monovalent cations from materials with nano-framework structures, as well as new intercalation compounds. The Element Reactivity Maps were also used to select barrier metals for device electrodes, and an autonomous contact resistance measurement system is under development.

## IMPACT STATEMENT

For materials informatics of chemistry-oriented search for new inorganic materials, we developed New Material Exploration Maps, Crystal Cluster Simulator, and Starrydata2 literature database, and carried out large-scale synthesis, ion-diffusion control and autonomous measurements.

## GRAPHICAL ABSTRACT

**Figure:**
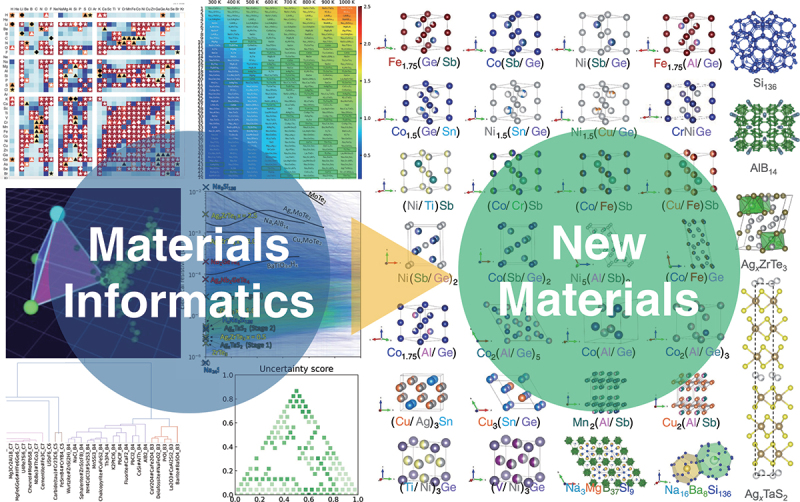

## 1. Introduction

Materials Informatics (MI), as part of the 4th paradigm of science (data-driven science), is increasingly recognized as a powerful tool for accelerating materials discovery [1–9]. In contrast, methods like first-principles calculations belong to the 3rd paradigm of science (computational science), which relies on simulations based on fundamental physical principles [10]. MI excels in identifying patterns and making predictions based on large datasets, which positions it as an effective approach for comparing materials. However, unlike the 3rd paradigm, MI does not directly depend on physical laws, meaning it often cannot produce a correct answer with a single prediction.

This difference creates challenges when integrating MI with traditional experimental research. MI is occasionally regarded as though it functions as a predictive technology, similar to first-principles calculations, and is often placed in the upstream stages of a physics-driven materials search. This approach misrepresents MI’s strengths, as it is not designed to deliver precise predictions in a single step, like computational physics-based methods. Instead, MI’s true potential lies in exploring broad chemical spaces and identifying trends or potential candidates for further investigation, making it a better fit for integration with chemistry-driven materials search.

In a physics-driven materials search, the process begins by selecting a specific application and formulating a physics-based hypothesis that is expected to lead to favorable properties for that application. A candidate material is chosen based on the hypothesis, and synthesis attempts are made, though these often result in unintended phases. The material’s properties are evaluated, and the synthesis is repeated under varying conditions until better properties than the reference are achieved. One advantage of this approach is that it provides a clear, hypothesis-driven plan that facilitates communication with funders. However, its low success rate is a significant challenge, as physics-based hypotheses and synthesis approaches often do not produce the desired results, leading to a low overall probability of success.

In contrast, the chemistry-driven materials search takes a broader exploratory approach by accepting the possibility of multiple applications. Instead of relying on a specific hypothesis, a chemical strategy is employed to enable the synthesis of new, unexplored materials. Once these materials are synthesized, their crystal structures are determined, and their features are investigated to identify potential applications. The material properties are then evaluated across several possible applications, and multiple targets are pursued in parallel, which increases the likelihood of success. The advantages of this approach include a higher success rate due to the increased number of samples and applications explored, along with the ability to produce scientific insights at various stages, leading to publications. Additionally, systematic experimentation generates valuable datasets that can be shared and cited by the scientific community.

By combining Materials Informatics with a chemistry-driven search, researchers can leverage MI’s strength in data-driven pattern recognition to explore vast chemical spaces and identify promising candidates for further study. This integration provides a more suitable and efficient path for materials discovery compared to placing MI within a physics-driven framework, where it may not be as effective.

In this context, we present our strategies for discovering new materials, which integrates experiments, theory, and data science. We have developed new MI methodologies for material exploration, including the New Material Exploration Map, the Crystal Structure Simulator, and the Starrydata database, which compiles experimental data curated from published papers. Additionally, we present the results of large-scale synthesis experiments and new materials exploration conducted using our proprietary high-throughput synthesis techniques. Finally, we showcase examples of MI applied to device fabrication, specifically in the search for barrier materials for electrode junctions in thermoelectric devices.

## 2. Our MI tools for new material exploration

**Figure 1.**
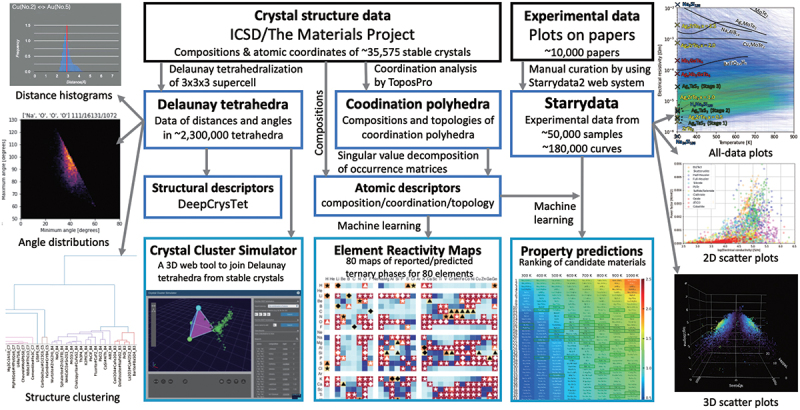
Overview of our original tools and analyses in materials informatics, developed for new materials search (containing an image from the Supplemental Material within reference [25]).

Figure 1 summarizes the representative MI technologies developed in our study. We used two types of data sources: external crystal structure data from Inorganic Crystal Structure Database (ICSD) [11] and the Materials Project [12], and experimental material property data independently collected from papers through Starrydata [13,14].

We performed Delaunay tetrahedral decomposition and coordination polyhedra analysis on CIF files of 35,746 stable crystal structures retrieved from the Materials Project [12], obtaining large-scale data on local crystal structures. Using these data, we created elemental descriptors and used them to predict compound formation possibilities through machine learning. Then we created a ‘New Material Exploration Map’ that illustrates the presence and formation probabilities of compounds for combinations of 80 × 80 × 80 elements. We developed a web tool called Crystal Cluster Simulator, which allows users to understand and connect Delaunay tetrahedra in 3D space using mouse operations. From the bulk analysis of Delaunay tetrahedral data, we attempted to organize and visualize various concepts of inorganic chemistry, such as chemical bonds and crystal structure prototypes.

Material property experimental data were collected manually from graphs in academic papers, by our data curators and anonymous users, using our independently developed Starrydata2 web system. Since thermoelectric materials were selected as the primary candidate application for the new materials, data curation focused on the temperature dependence of thermoelectric properties. Analyses using scatter plots and property predictions through machine learning also focused on thermoelectric properties.

### 2.1. Prediction of phase formation probability

When investigating the discovery of past inorganic compounds, we find that some combinations of elements have already been reported, while others have not yet been found. For those not yet found, there are two possible cases: either the elements do not react despite efforts, or they have not been studied due to a lack of attention from past researchers. The latter case are the promising targets for new material exploration. By applying machine learning to the reactivity of elements and considering the similarities in their behaviours within crystal structures, we can predict which combinations of elements are likely to react and form ternary phases. Once we predict the possibility of forming some kind of multicomponent compound, regardless of the crystal structure, we can focus on synthesis experiments with these combinations of elements. After determining the crystal structures of the obtained new compounds, we will be able to suggest the application of the new compound.

#### 2.1.1. Atomic descriptors from coordination polyhedra

Element descriptors are numerical vectors (sets of numbers) used in machine learning programs to represent the characteristics of elements. Popular element descriptors, such as those from Pymatgen [15] and XenonPy [16], use data on the physical properties of elements. These include basic data of each element, such as atomic number, mass number, and group number, as well as intrinsic values like ionic radius and electronegativity. Physical property values of crystals of pure elements such as melting point, boiling point, and mechanical properties are also included, although they may not be transferable in crystals of different bonding characters. Instead, more information on the local crystal structures around each element should be included to discuss about the formation probability of crystals.

Zhou et al. developed lightweight element descriptors named Atom2vec, by performing singular value decomposition on the occurrence frequency matrices of partial chemical formula [17]. However, Atom2vec did not account for the local structures around the constituent element.

We developed new element descriptors based on local crystal structures [18]. We extracted coordination polyhedra around each atom in the crystal structures of stable compounds using ToposPro [19]. We created occurrence matrices from the number and types of coordinating elements, and occurrence matrices for the topology of the coordinating elements. Each matrix has 80 rows (one for each central element) and *N* columns (one for each environment type). For each combination of central element and environment type, a value of 1 indicates the combination exists in the dataset, and 0 indicates it does not. We defined environment types in three ways: by overall chemical composition (CMP, to replicate Atom2vec [17], by coordinating elements and their number in the coordination polyhedra (CRD), and by the topology of the coordination polyhedra (TPL). In all cases, *N* is much larger than 80.

We use the product of the first and second matrices from the Singular Value Decomposition (SVD) as the element descriptors. The first 80 × 80 matrix represents the characteristics of the 80 elements through its left singular vectors, while the second 80 × *N* matrix contains the singular values, which serve as weights for these descriptors. The 80 × 80 section on the left side of this matrix forms a diagonal matrix, while the entire right-side section, consisting of 80 × (*N* − 80) entries, results in zeros as an outcome of the SVD process. The third *N* × *N* matrix, which is not used in this context, describes the element behaviors in terms of their crystallization trends, such as composition, coordinating elements, and coordinating topology. When multiplying the first two matrices, we obtain a weighted 80×*N* diagonal matrix. From this product, we extracted the 80 × 80 portion, where each row corresponds to an element, and each column represents a specific feature.

These extracted features were used as element descriptors in further analyses. By selecting a column and displaying the scalar values of the feature for each element in a periodic table format, we observed that some features resemble known properties such as metallicity, ionic size, and electronegativity. We refer to the row for each element (an 80 × 1 vector, or a 240 × 1 vector when combined with additional descriptors) as an ‘element vector’. The inner product of two element vectors allows us to quantify the similarity between the corresponding elements.

#### 2.1.2. New material exploration maps

We used these element descriptors to perform machine learning on the formation possibility of ternary compounds, and compiled the results as New Material Exploration Maps (or Elemental Reactivity Maps) [20]. By training the machine learning model with systems where known ternary compounds exist (labeled as 1) and those where they do not (labeled as 0), we constructed a neural network that predicts the formation possibility of ternary compounds for each combination of elements as a value between 0 and 1. We summarized these predictions in a grid format to create a new material exploration map.

Considering ternary combinations of 80 elements available in the laboratory, we outputted 80 maps consisting of 80 × 80 grids. Systems with reported compounds were shown in shades of red, and systems without reported compounds in shades of blue. The probability of compound formation was represented by the intensity of the colors. The reliabilities of the structural data were displayed with symbols. and choose promising systems for their experiments.

For example, absence of reported ternary compound and high probability of new compound synthesis is displayed in dark blue. Red squares indicate the presence of existing compounds, but there may still be undiscovered new compounds so these systems should also be considered for parallel experimentation. Selecting light blue squares can help identify elements less likely to react with the target compound. For example, in crystal growth using the flux method, this information can be used to select flux elements that are less likely to react with the target compound.

### 2.2. Delaunay Tetrahedralization for structural chemistry

#### 2.2.1. Delaunay Tetrahedron Database

Delaunay triangulation is a classical mathematical method of partitioning space using a set of arbitrary points as vertices [21]. In 2D, this method partitions a set of points into triangles (Delaunay triangulation), and in 3D, into tetrahedra (Delaunay tetrahedralization). While there are numerous ways to partition points into triangles or tetrahedra, Delaunay triangulation ensures that no other vertices lie inside the circumcircles of the triangles or circumpheres of the tetrahedra. This results in a nearly unique partitioning of space without gaps, except in highly symmetrical configurations like squares or cubes. The chosen triangles and tetrahedra tend to be as close to equilateral triangles and regular tetrahedra as possible.

Delaunay triangulation has long been used in computer graphics, but rarely applied in crystal chemistry [22,23]. However, its complementary method, Voronoi partitioning, has been widely used. A Voronoi polyhedron is formed by the perpendicular bisectors of the edges of Delaunay tetrahedra and represents a complex polyhedron formed around a vertex. When trying to understand crystal structures as tessellations of spatial shapes, it is more practical to assemble simple Delaunay tetrahedra than complex Voronoi polyhedra. Since the information obtainable from Delaunay tetrahedra remains unexplored, there is potential to gather useful information for crystal structure MI from them.

In this study, we developed a program to perform Delaunay tetrahedralization on supercells of known stable compounds included in the Materials Project. We evaluated the number of independent Delaunay tetrahedra present within the crystal structures. We extracted the shapes, angles, and edge lengths of these tetrahedra and compiled them into a Delaunay Tetrahedron Database for use in MI. Although this database is not open, similar results of the Delaunay tetrahedralization for each crystal structure are published with an interactive web interface [24].

#### 2.2.2. Delaunay Chemistry

We attempted to visualize the chemical properties of each tetrahedron by using the Delaunay Tetrahedron Database with the constituent elements. From the distribution of the shortest and longest edges, we extracted information corresponding to the valences of each ion. From the distribution of the smallest and largest angles, we found the potential to extract information about the nature of chemical bonds. By outputting these on the periodic table, we aimed to use it as a resource for learning the vast information of inorganic chemistry.

Additionally, we performed Delaunay tetrahedralization on 100 representative crystal structure prototypes from the ICSD database and clustered them using Ward’s method to create a dendrogram [25]. As a result, crystal structures were classified into seven major branches, successfully visualizing the similarities of crystal structures based on local structures, such as the branch of transition metal oxides.

We also conducted research using Delaunay tetrahedra as crystal structure descriptors for machine learning [26]. This method, named DeepCrysTet, was utilized in machine learning and confirmed to provide excellent predictions as an alternative to existing methods that represent atomic bonds as graph networks.

#### 2.2.3. Crystal cluster simulator

Using the Delaunay Tetrahedron Database, we developed the Crystal Cluster Simulator [27]. This tool allows users to manually assemble clusters of crystal structures based on known stable compounds by connecting atomic tetrahedra at their triangular faces.

**Figure 2.**
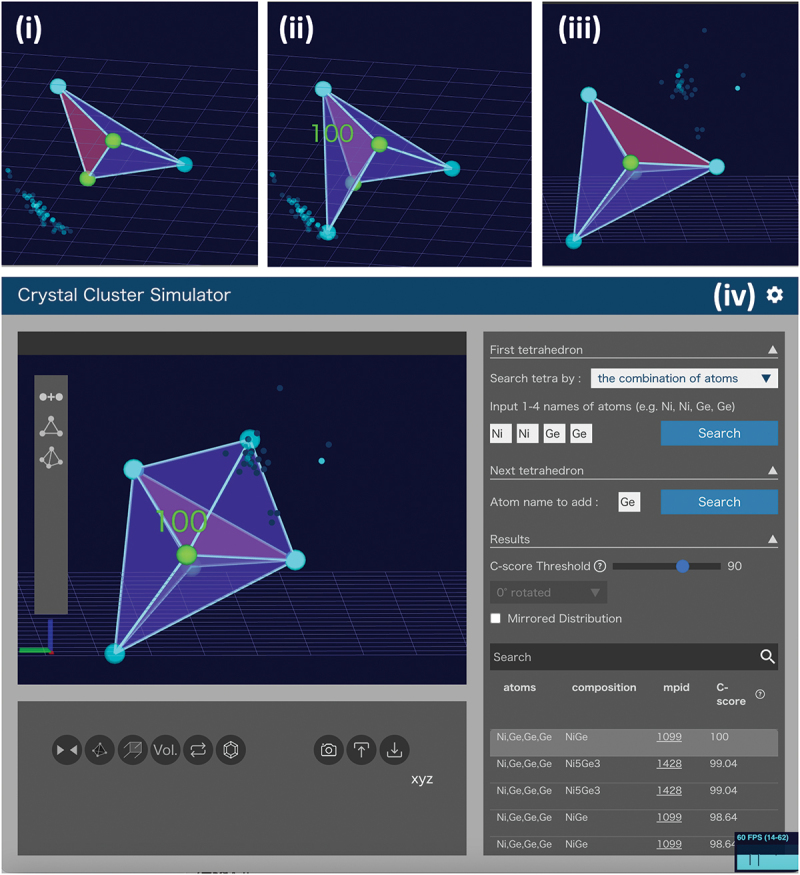
Crystal cluster building based on atomic coordinate predictions using Delaunay tetrahedra in stable crystals, implemented in the crystal cluster simulator.

Figure 2 illustrates an example of its operation. To prototype possible crystal structures of Ni-Ge compounds, we first searched the database for Delaunay tetrahedra with the composition Ni-Ni-Ge-Ge. Figure 2(i) shows a state where one of the tetrahedra from the NiGe compound is selected and placed. Here, we selected one triangular face, Ni-Ni-Ge, and displayed candidate coordinates for adding a new Ge atom. Each point represents a prediction from one Delaunay tetrahedron. Selecting one of these points displays the original compound (NiGe) with a matching score (100%) based on the triangular shape, as shown in Figure 2(ii). Figure 2(iii) shows a state where a new tetrahedron, manually added with already displayed atoms, is selected for another triangular face, Ni-Ge-Ge, to predict new coordinates for adding Ge. Selecting a point adds a new tetrahedron, as shown in Figure 2(iv). By repeating this process, clusters of local crystal structures can be assembled. This tool may be a crystal structure prediction tool requiring substantial cognitive effort. However, the reasoning and considerations researchers engage in while assembling known crystal structures deepen their understanding of crystal structures. Despite appearing simple, many points require careful thought, such as when connecting an atom at a specific site prevents further connections. This process would mirror actual crystal growth, and researchers may develop a direct understanding of crystal structures through this puzzle-like practice.

Most existing crystal structure visualization softwares like VESTA [28] requires atomic coordinates to be input as fractional coordinates with specified space groups and lattice constants, making intuitive understanding difficult. Additionally, editing known crystal structures and comparing local structures with other stable crystal structures is challenging when placing new atoms. This tool allows intuitive editing of crystal structures using mouse dragging. Users can rotate atomic clusters to align desired planes with the computer screen and drag atoms accordingly. Bond lengths compared to known compounds are displayed with colors, allowing users to assemble structures within a natural range.

To verify the periodicity of the assembled cluster, users can select two atoms of the same element to define three unit-cell vectors. This enables the display of the repeated spatial structure, confirming boundary matching. Users can also load and edit crystal structures output in XYZ format by external crystal structure prediction tools to design new crystal structures.

The Crystal Cluster Simulator will implement features for creating input files for first-principles calculations and molecular dynamics simulations. For instance, researchers can consider reaction pathways while imagining adsorption patterns and migration paths of molecules on crystal surfaces. To support this, the tool provides features for adding and independently moving and rotating molecules. This can facilitate discussions among chemical researchers and enable them to create input files for molecular dynamics simulations, potentially making catalyst development more active.

### 2.3. Starrydata: experimental data from literature

#### 2.3.1. Starrydata2 web system

In many fields of functional materials science, overviewing most of the past attempted experiments is challenging. Additionally, much of the experimental data obtained from past research is difficult to access in digital format, making it hard to apply the latest MI technologies, such as machine learning.

Therefore, we established an open academic platform named Starrydata, for large-scale collection of experimental data from plots in academic papers [13]. The Starrydata2 web system [14] manages papers based on their DOIs and records numerical data extracted from plot images along with sample information linked to each paper. The user interface is like a bibliographic database but allows sharing data without sharing any copyrighted paper PDFs or plot images. The users can contribute to extract data from published papers, and the uploaded data are shared to all users. As more researchers use Starrydata as a repository for raw data from plots in papers, the database grows larger.

Data collection from plot images requires complex and adaptive interpretation skills. Thus, we obtain high-quality data manually through curators rather than full automation. However, we are developing tools to accelerate this work, including tools for entering sample information using large language models.

The data collection fields of Starrydata cover a wide range of functional materials such as thermoelectric materials, magnetic materials, and battery materials, as well as fundamental research areas like solid-state physics and quasicrystals. We plan to add new data collection projects as we receive requests for collaborative research. Currently, the focus is on inorganic materials, but we believe it is possible to target data collection in any research field where many experimental data are reported as template-like plots.

In Starrydata’s data collection, we register experimental data for any original sample from the paper, increasing the data volume. Instead of reading out single values from the curves, we collect the entire curve as numerical data and use interpolation to extract arbitrary values programmatically. This approach enabled us to build very large thermoelectric property database, registering approximately 50,000 thermoelectric material samples.

The data collected by Starrydata is published as open data, available for use by both commercial and non-commercial entities for citation only. Researchers and engineers worldwide are allowed to analyze this data from different approaches, leading to new discoveries and tool development.

#### 2.3.2. Analyses of the starrydata dataset

Examples of analyses using large-scale experimental data on thermoelectric properties are shown on the right side of Figure 1. In the All-data plot (also shown in Figure 5(iii)), we visualized the relationship between the temperature dependence of electrical resistivity, color-coding tens of thousands of curves based on maximum *ZT* values. This allowed experimental researchers to estimate the potential of thermoelectric material samples just by measuring electrical resistivity, without spending days on the cumbersome and time-consuming measurement of thermoelectric properties.

As an example of a two-dimensional scatter plot, we showed the relationship between the power factor and electrical conductivity at room temperature. Similar interactive scatter plots can be created by users with the Starrydata Sample Explorer [29]. The inverse Jonker plot, with the Seebeck coefficient SSS on the horizontal axis and the logarithm of electrical conductivity log *σ* on the vertical axis, is particularly useful for optimizing thermoelectric properties. It visualizes the common *S* and log σ regions where high *ZT* materials manifest.

In Starrydata Explorer 3D, we also provide a three-dimensional interactive scatter plot with *S*, log *σ*, and lattice thermal conductivity *κ*_L_ as axes. By rotating and viewing this starry-sky-like plot, researchers can conduct comprehensive materials science research.

#### 2.3.3. Machine learning of thermoelectric properties

We represented the chemical composition of samples recorded in Starrydata using element descriptors and trained the model with *ZT* values at 100 K intervals as training data. Subsequently, we used this machine learning model to predict *ZT* at various temperatures for approximately 7,000 semiconductors recorded in the Materials Project. By ranking these predictions in descending order of predicted *ZT* values, we could list chemical compositions that appear promising as thermoelectric materials. Evaluating the prediction accuracies of this table is challenging, because the properties of thermoelectric materials with the same chemical composition can vary significantly as shown in the All-data plot. Moreover, until long-term optimization experiments such as carrier doping are conducted, it is unclear how high the *ZT* of the material can reach experimentally. Improvement of the machine learning techniques considering the effects of the data bias is in progress [30].

Such a list of promising compounds can help convey the experience and intuition of veteran thermoelectric materials researchers to experimental researchers who are still unfamiliar with thermoelectric materials. Many compositions listed in the table are intuitively perceived as ‘thermoelectric material-like’ even by experienced researchers. We hope experimental researchers will find compounds that are interesting and feasible to synthesize by themselves.

## 3. Experimental searches for new materials

### 3.1. Large-scale sample synthesis

By considering the ranking of the predicted *ZT* values and the new material exploration map, we selected combinations of three elements with a high possibility of yielding new compounds with expected good thermoelectric properties. We conducted large-scale synthesis experiments on combinations of many elements through direct reactions of each element, without setting a specific target crystal structure.

#### 3.1.1. Systematic synthesis and characterization

**Figure 3.**
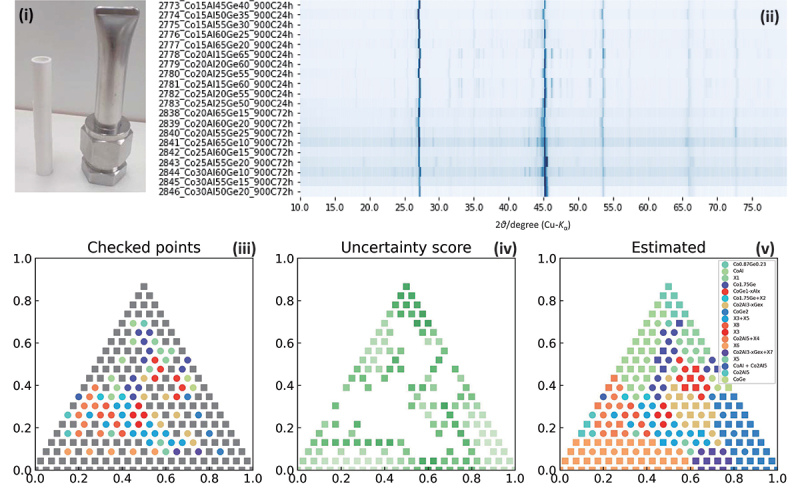
Overview of large-scale sample synthesis experiments. (i) SUS reaction container with three BN crucibles containing pelletized raw powder. (ii) comparison of multiple XRD patterns in a color map like debye-scherrer photograph. (iii) experimental results of the combination of the phases, (iv) uncertainty scores of unexplored chemical compositions, and the (v) predicted ternary phase diagram, given by the machine-learning-based phase diagram AIPHAD[31].

Figure 3 displays our chemistry-driven methods for large-scale synthesis experiments involving many samples. We selected systematic combinations of elements that frequently contain dark blue cells on the new material exploration map. Referring to systems where many promising thermoelectric materials have been found, we included elements such as Si, Ge, and Sb. We focused on two metal elements that are easy to handle in the laboratory as powders, mixed in six molar ratios. These were mixed in an inert atmosphere using a mortar in air, then pressed into pellets and placed in BN crucibles, as shown in Figure 3(i). In an Ar-gas filled glovebox, these were set in triplicate and sealed in a custom stainless-steel container that can be sealed just by screwing. The containers were placed in an electric furnace and heat-treated typically at 1173 K in the air, allowing for solid-phase and solid-liquid reactions. The containers were opened in air, and the samples were pulverized to measure powder X-Ray Diffraction (XRD) patterns by using D2 PHASER (Bruker, Germany). To assist finding the common sets of unknown peaks, we developed a web tool to display multiple powder XRD patterns as color intensity maps like Debye-Scherrer photographs, as shown in Figure 3(ii). When unknown phases were observed, we evaluated their chemical compositions through Energy Dispersion X-ray spectroscopy in a Scanning Electron Microscope (SEM-EDX).

**Figure 4.**
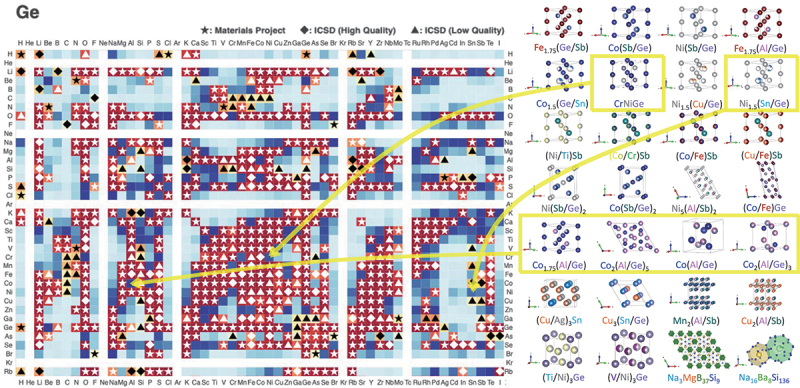
Element reactivity map for ternary systems containing Ge and newly discovered ternary compounds from large-scale synthesis experiments.

Among the unknown phases, those with easily determined crystal structures were solid solutions formed by partial element substitution of known compounds. These are illustrated in Figure 4 along with the new material exploration map. We regarded solid solutions of known crystal structures as new materials, if they are combinations of elements not reported in crystal structure databases like ICSD. By varying the starting composition and heat treatment conditions, we attempted to increase the yield of new phases. When single crystals at the tens of micrometer level were obtained, we refined the structural parameters using single-crystal XRD. Although many unreported samples showed more complex powder XRD patterns with unknown peaks, suggesting the generation of new materials with highly novel crystal structures, which we aim to determine.

In Figure 4, ternary systems with yellow borders are combinations of elements shown in dark blue on the new material exploration map. Notably, in the Co-Al-Ge ternary system, which was shown in very dark blue, four new compounds were found. These results demonstrate the effectiveness these maps for the new material exploration approach.

In ternary systems where the presence of new materials was indicated, we conducted additional experiments and attempted to efficiently optimize the composition using the machine-learning-based phase diagram system AiPHAD [31,32]. This process is shown in Figures 4(v–vii). We displayed regions forming the same set of compounds in the same color. By label-propagation method to evaluate the uncertainty score, we proposed initial compositions with high uncertainty for the next experimental conditions. By repeating such synthesis experiments, we succeeded in the synthesis and determination of the crystal structures of the four new phases. We successfully obtained the phase diagram of the Co-Al-Ge ternary system, including four newly identified phases. Initially, we synthesized 6 samples for this system and found that all of them contained unknown phases. We then synthesized 89 additional samples at compositions suggested by AIPHAD to pinpoint the compositions of the four new phases. The synthesis and evaluation of these 95 samples were made easy by our high-throughput synthesis methodology.

### 3.2. New material with novel crystal structure

The metal containers used in large-scale synthesis experiments were originally designed for chemical reactions involving volatile and highly reactive elements like alkali metals. We developed a unique synthesis method called reaction sintering via Na solution [33–35], where B powder is impregnated with a solution of Na and Si, promoting simultaneous reaction and sintering. We succeed to obtain bulk samples of known compound Na_8_B_74.5_Si_17.5_ for the first time as a bulk [36], and achieved *ZT* = 0.08 at 973 K.

We developed Na-Mg vapor supply method, and succeeded to synthesize a new compound Na_3_MgB_37_Si_9_ with a novel crystal structure [37]. While Si forms a one-dimensional chain in Na_8_B_74.5_Si_17.5_, a layered network of B is formed in Na_3_MgB_37_Si_9_. During the single crystal XRD analysis, the elements occupying a crystallographic site was not identified. So we used the Crystal Cluster Simulator, to evaluate the validity of each element based on reported Delaunay tetrahedra. We found that Mg, rather than the initially assumed Si, were likely to occupy the site. This new material achieved *ZT* = 0.13 at 973 K, although we are exploring other application fields for this compound.

### 3.3. New ion diffusion control techniques under electric field

Like in the previous section, the development of new synthesis methods is a crucial approach for discovering unexplored new materials. Particularly, if there are methods to selectively insert or remove specific ions in the crystal structure, it allows for the design of new materials from a desired property, potentially leading to the synthesis of many promising compounds.

#### 3.3.1. Ion-control synthesis methods

**Figure 5.**
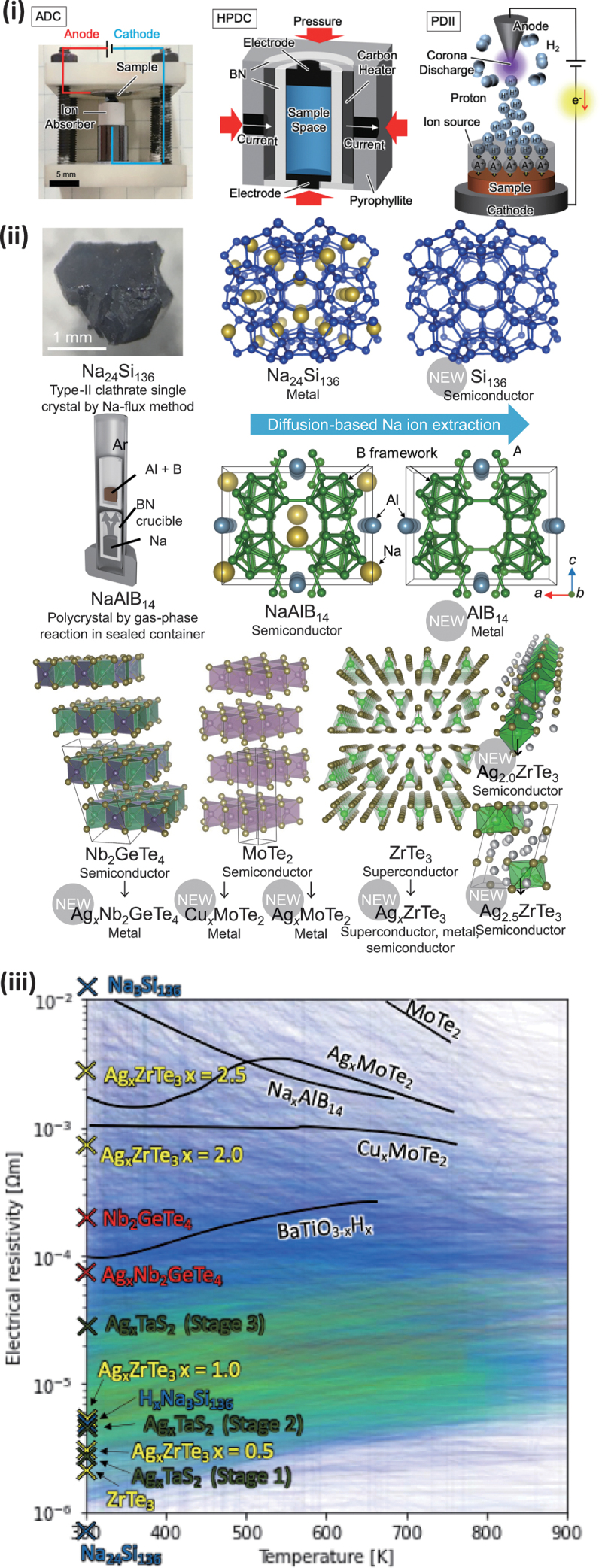
(i) Schematics of the proprietary ion diffusion control techniques. (ii) examples of synthesized new materials. (iii) comparison of the electrical resistivity of synthesized samples with the All-data plot from Starrydata. Data in Starrydata are color-coded in blue, green, and red, indicating increasing maximum *ZT*. Reproduced by permission from [38, 39, 51], copyright 2022, 2023, John Wiley and Sones, American Chemical Society.

Figure 5 shows the new synthesis methods we developed. Anisotropic Diffusion Control (ADC) is a synthesis method that changes chemical composition by diffusing ions in a solid using electric fields or chemical potential gradients [38]. High Pressure Diffusion Control (HPDC) involves conducting ADC under high pressure, allowing simultaneous control of temperature, pressure, and voltage [39]. This method applies constant pressure to counteract volume changes associated with chemical composition changes, preventing cracks and enabling efficient ion removal and insertion [40]. Proton Driven Ion Introduction (PDII) uses H^+^ ions generated by corona discharge in hydrogen gas to irradiate a solid electrolyte, pushing out conductive ion species in the solid electrolyte to compensate for charge neutrality and absorb them into the sample [41–43].

#### 3.3.2. Na^+^ extraction from Type-ii Si clathrates

Figure 5 illustrates compounds successfully synthesized using these methods. Na_24_Si_136_, a compound with a Type-II clathrate structure, contains Na^+^ ions within a cage-like Si network. Removing Na^+^ is expected to cause significant changes in electronic properties, transitioning from metallic to semiconductor behavior. However, previous successful Na^+^ removal was limited to powdered samples with grain boundaries [44]. Evaluation of intrinsic physical properties requires homogeneous Na^+^ removal from bulk single crystals.

In this study, we succeeded in forming Si clathrate single crystals with Na^+^ occupancy reduced to 7% [45] by removing Na^+^ while maintaining the Si framework from the world’s largest Na_24_Si_136_ clathrate single crystal [46–48] made by the Na-Sn flux method. The electrical resistivity increased by four orders of magnitude at room temperature, confirming the transition from metallic to semiconductor behavior. Further optimization of carrier concentration may make thermoelectric applications feasible. Additionally, electronic structure calculations suggest that empty Si_136_ is a direct transition semiconductor, potentially more suitable for solar cell applications than the indirect transition semiconductor diamond-structured Si. Additionally, we succeeded in synthesizing stoichiometric Na_16_Ba_8_Si_136_ Type-II clathrate for the first time, while Na-deficient crystal with the composition Na_9.8_Ba_8_Si_136_ has been reported [49].

#### 3.3.3. Na^+^ extraction from NaAlB_14_

In the thermoelectric property prediction ranking obtained from the machine learning analysis of Starrydata introduced in Figure 1, one of the top-ranked materials was NaAlB_14_. This semiconductor contains Na^+^ within a covalent B framework. Like Type-II clathrates, this has a one-dimensional zigzag tunnel structure that could serve as a conduction path for Na^+^ ions. Although this compound is already known, previous reports were limited to microcrystals obtained by Al-flux method [50], and its electronic properties had not been reported.

In this study, we optimized the solid-phase reaction process in Na vapor. As illustrated in the middle part of Figure 5, we successfully achieved the single-phase bulk polycrystal of NaAlB_14_ for the first time [51]. While it possessed semiconducting electrical conduction, we observed that it systematically transitions to metallic conduction upon Na^+^ ion removal. Using HPDC, we successfully synthesized a new material, AlB_14_, with complete Na^+^ removal [39]. We are currently exploring new functionalities for this material.

#### 3.3.4. Cu^+^/Ag^+^ intercalation to chalcogenides

These ion diffusion control techniques can also be applied to intercalation of monovalent ions into layered materials and quasi-one-dimensional structures bonded by van der Waals forces, as shown in the lower part of Figure 5(ii). Machine learning with Starrydata suggested high thermoelectric properties in Te compounds, guiding us to narrow down target materials containing Te for intercalation. Using HPDC, we successfully synthesized a new material by introducing Ag^+^ ions into Nb_2_GeTe_4_. This transformed Nb_2_GeTe_4_ from a semiconductor to a metal, reducing the room-temperature electrical resistivity from 2 × 10^−2^ Ωcm to 6 × 10^−3^ Ωcm. We also successfully intercalated Cu^+^ and Ag^+^ into the layered semiconductor MoTe_2_, achieving nearly two orders of magnitude reduction in room-temperature electrical resistivity. However, the distribution of high *ZT* samples in the All-data plot of Starrydata indicated the need for an additional order of magnitude reduction in electrical resistivity.

Using PDII, we introduced Ag^+^ ions into single crystals of the quasi-one-dimensional metal ZrTe_3_, synthesizing the new material Ag_*x*_ZrTe_3_. Transmission Electron Microscopy revealed that intercalating Ag^+^ into the needle-like ZrTe_3_ single crystals, obtained by vapor synthesis, gradually amorphized the crystal while maintaining its external shape. This is likely due to the ZrTe_6_ trigonal prisms transforming into octahedra with increasing Ag_+_ content. ZrTe_3_ is reported to exhibit filamentary superconductivity with a critical temperature *T*_c_(onset) = 1.7 K. We discovered that Ag^+^ intercalation increased *T*_c_ to 6.3 K in crystals with *x* = 0.5 [52]. Furthermore, in crystals with higher *x*, the electrical resistivity continuously increased, transforming the material into a semiconductor. Evaluation of thermoelectric properties is challenging due to the needle-like crystal shapes. However, possibly low *κ*_L_ due to the amorphous structure and the broad range of electrical resistivity covering the high-ZT region in Figure 5(iii) suggest the potential for synthesizing high *ZT* crystals of this compound.

## 4. MI for thermoelectric device fabrication

The new material exploration map can also be used to predict combinations of elements with low reactivity. In thermoelectric conversion devices, metal electrodes are joined to thermoelectric materials and used for long periods at high temperatures. During this time, metals like Cu used in the electrodes diffuse and react with the main thermoelectric material (such as Bi_2_Te_3_), causing degradation. Although Ni metal has long been used as a barrier metal, it has the problem of insufficient diffusion suppression. Using the new material exploration map, we selected metals predicted to have lower reactivity with the elements composing common thermoelectric semiconductors Bi, Te, and Sb, compared to Ni.

These metals were sputter-deposited on the surface of 3 mm-square (1 mm thick) thermoelectric semiconductor chips (p-type: (Bi_0.15_Sb_0.85_)_2_Te_3_, n-type: Bi_2_Te_3_) to form films approximately 100–500 nm thick. Durability tests were conducted at 150°C in air for 100 hours. The results showed that the currently used Ni reacted strongly with the n-type Bi_2_Te_3_. However, for the barrier metals Ti, Cr, and V, almost no discoloration due to reactions was observed with both the p-type (Bi_0.15_Sb_0.85_)_2_Te_3_ and the n-type Bi_2_Te_3_. Therefore, these metals were suggested as promising as new barrier materials for electrodes on Bi_2_Te_3_-based thermoelectric modules.

**Figure 6.**
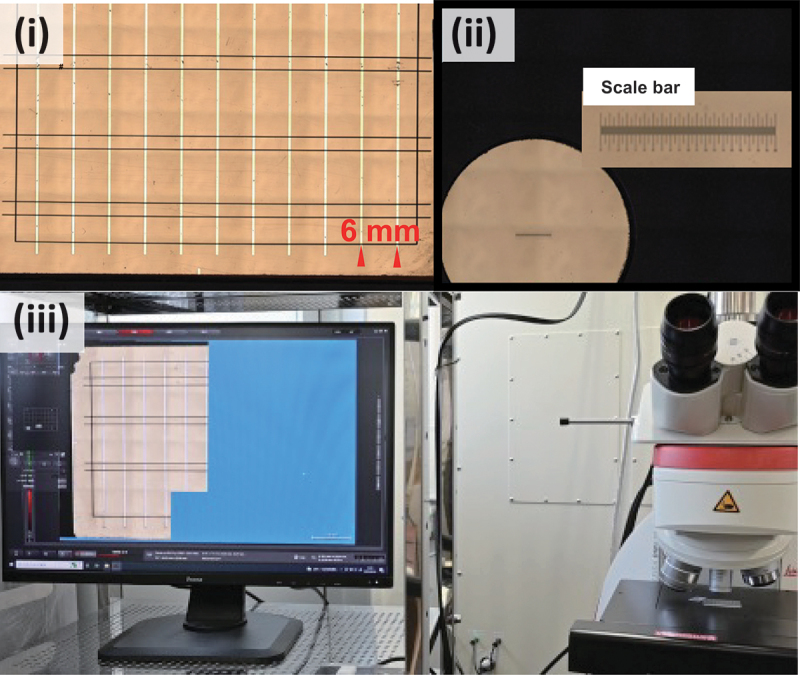
Autonomous measurement system for contact resistance by the transfer line method (TLM). (a) Sample for measurement, with metal films and electrodes deposited on a semiconductor film. (b) Circular region for automatic recognition of the scale bar. (c) scale bar. (iv) automatic scanning of a target sample by computer-controlled sample stage and optical microscope.

For a more fundamental design of the electrode metallization (junction structure), it is necessary to measure the contact resistance of the electrodes rather than qualitative experiments like observing discoloration. However, the Transmission Line Model (TLM) method, widely used for evaluating contact resistance, is cumbersome and time-consuming to prepare measurement samples. Therefore, in this study, we developed an apparatus to extend TLM measurements for high-throughput testing. Figure 6 shows the autonomous measurement system under development. A thermoelectric material thin film is deposited on an SiO_2_ substrate, and a metal thin film is deposited in the pattern shown in the photo. By controlling an optical microscope and sample stage with a computer, we developed an apparatus that performs image recognition and automatic measurement using a custom Python script. We have successfully automated the determination of probe placement and the measurement of distances between terminals. Once completed, this apparatus is expected to obtain large-scale contact resistance data for numerous samples with varying thermoelectric materials, metal elements, and film thicknesses.

## 5. Summary

We introduced our Chemistry-oriented research style, in which various MI tools are used by experimentalists to select the candidates, and the materials functions are determined after synthesizing new materials.

We developed our own MI methodologies to support this new material exploration. Based on 80 new material exploration maps that summarize the distribution of known compounds and the predicted probabilities of compound formation, we aimed to systematize a new inorganic chemistry based on Delaunay tetrahedral decomposition of crystal structures. Additionally, we developed a Crystal Cluster Simulator using this data, aiming for an intuitive and interactive new crystal structure design visualizer while deepening the understanding of inorganic structural chemistry. In the Starrydata project, we collected large-scale experimental data from graphs in papers, building an environment for a comprehensive exploration of functional materials such as thermoelectric materials through plots and machine learning.

Sharing these insights with experimental researchers with unique experimental techniques enabled new material exploration through large-scale synthesis experiments and ion diffusion control. We successfully synthesized solid solutions of many known compounds, new borides with novel structures, and compounds with ions removed from cage-like compounds. By comparing these materials with past experimental data recorded in Starrydata, we speeded up the new material exploration research without being tied to the investigation of a single material for too long. Using techniques for visualizing large amounts of published experimental data and proposing the next experimental conditions based on data science, we succeeded to discover more than 30 new materials, and some of them are under investigation of applications.

We believe that intuitive databases and tools that assist researchers’ thinking are effective for enhancing the efficiency of global materials science. By openly sharing our research style, developed tools, and datasets with researchers worldwide, we hope to improve the efficiency of materials science and facilitate the discovery of many new materials.

## Statement of Novelty

For materials informatics of chemistry-oriented search for new inorganic materials, we developed New Material Exploration Maps, Crystal Cluster Simulator, and Starrydata2 literature database, and carried out large-scale synthesis, ion-diffusion control and autonomous measurements
